# Iatrogenic Iron Overload Causing Porphyria Cutanea Tarda in a Patient With a Rare Nonsense Heterozygous UROD Gene Mutation

**DOI:** 10.7759/cureus.16215

**Published:** 2021-07-06

**Authors:** Hallie B Anderson, Michael H Storandt, Rekha Kallamadi, Dinesh Bande, Abhishek Matta

**Affiliations:** 1 Internal Medicine, University of North Dakota School of Medicine and Health Sciences, Grand Forks, USA; 2 Internal Medicine, Sanford Health, Fargo, USA; 3 Hospital Medicine, Sanford Health, Fargo, USA

**Keywords:** porphyria cutanea tarda, hemochromatosis, urod mutation

## Abstract

Porphyria cutanea tarda (PCT) is a rare dermatologic condition characterized by blistering of sun-exposed surfaces and elevated hepatic enzymes. It may infrequently occur as the primary presentation of underlying hemochromatosis. A 61-year-old female with anemia caused by chronic kidney disease and end-stage renal disease on hemodialysis presented with a bullous rash on her hands with associated pruritus. The rash worsened despite conservative treatment. An initial biopsy demonstrated a pauci-inflammatory cell-poor subepidermal cleft. Subsequent workup revealed elevated serum and urine porphyrins, confirming a diagnosis of PCT. Additionally, her skin was darkened and ferritin was elevated. MRI of the liver demonstrated iron overload with genetic testing negative for *C282Y* or *H63D* mutations, supporting a diagnosis of secondary hemochromatosis. Further genetic testing revealed that the patient had a rare heterozygous nonsense mutation of the *uroporphyrinogen decarboxylase* (*UROD*) gene, for a sequence variant designated c.616C>T, which is predicted to result in premature protein termination (p.Gln206*). PCT occurs due to decreased function of *UROD*, leading to accumulation of porphyrins causing dermatologic manifestations and liver injury. *UROD* is inactivated in an iron-dependent process, explaining the mechanistic link between hemochromatosis and PCT.

## Introduction

Porphyria cutanea tarda (PCT) is caused by a hepatic deficiency of uroporphyrinogen decarboxylase (UROD), an enzyme responsible for catalyzing the conversion of uropophyrinogen to coproporphyrinogen in the heme synthesis pathway. UROD deficiency leads to the accumulation of uroporphyrinogen, which is oxidized to form uroporphyrins. When exposed to sunlight, these uroporphyrins cause an immune-mediated reaction and the release of free radicals, causing the characteristic blistering lesions of PCT [1]. Most cases of PCT are classified as sporadic and are caused by viruses and substances that interfere with hepatic function, such as the hepatitis C virus, human immunodeficiency virus, high alcohol consumption, and iron overload [2]. Less commonly, familial mutations in the *UROD* gene can cause an increased risk of PCT in conjunction with substances that reduce UROD enzyme activity [1,2]. Here, we present a case of PCT caused by an iatrogenic iron overload due to frequent iron infusions for anemia caused by chronic kidney disease (CKD) in the setting of a rare heterozygous *UROD* mutation.

## Case presentation

**Figure 1:**
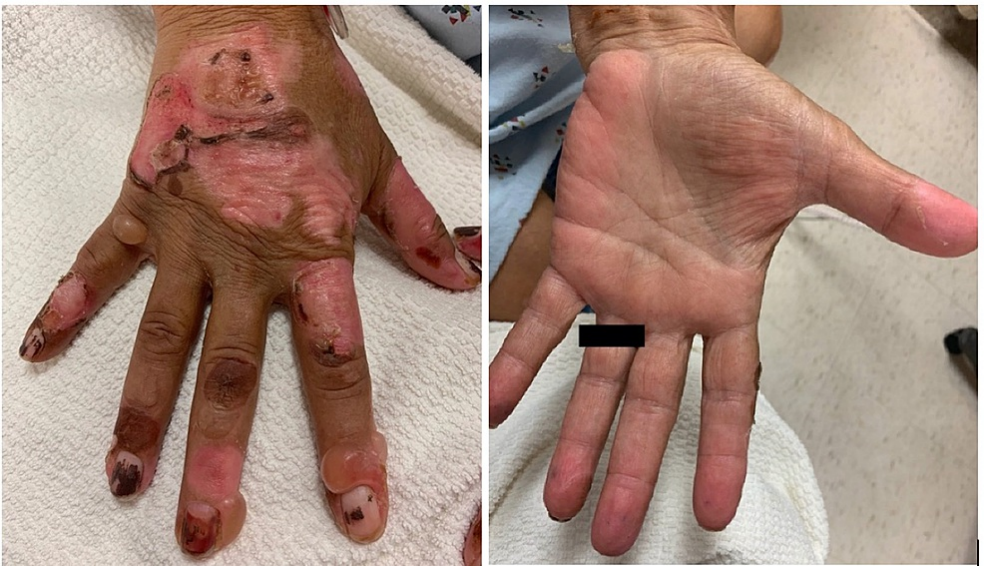
Image of the hand demonstrating multiple bullae with sparing of the palms.

A 61-year-old female with a past medical history of anemia caused by CKD and end-stage renal disease (ESRD) on hemodialysis presented to the emergency room with acute-onset blisters on the dorsum of her hands, sparing her palms, with associated pruritus. Her pre-dialysis creatinine levels ranged from 5.99 to 8.62 mg/dL, and her red blood cell indices ranged from 2.42 to 2.62 M/uL in the month prior to the initial presentation. She was started on conservative treatment with prednisone, diphenhydramine, and triamcinolone cream. Three weeks later, the patient presented to the emergency department again for worsening of the rash (Figure 1) along with facial swelling. She was admitted, and clinical genetics and hematology-oncology were consulted.

**Figure 2:**
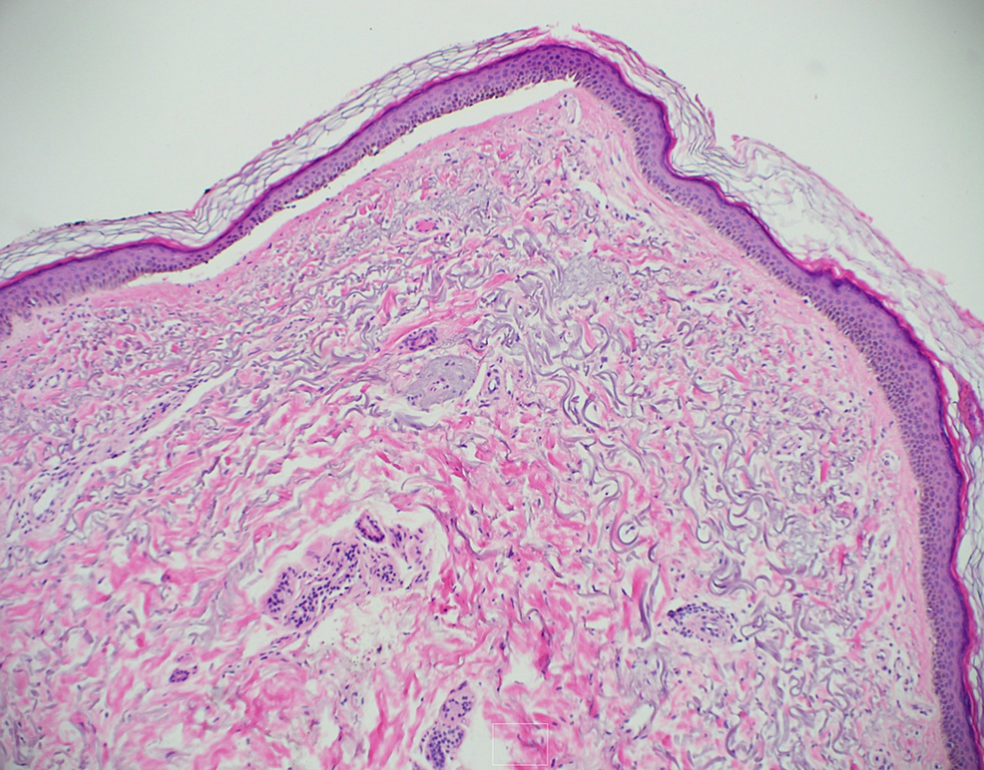
Biopsy showing thick homogeneous deposition of IgG, IgA, and fibrinogen within the walls of superficial dermal vessels. Ig: immunoglobulin

**Figure 3:**
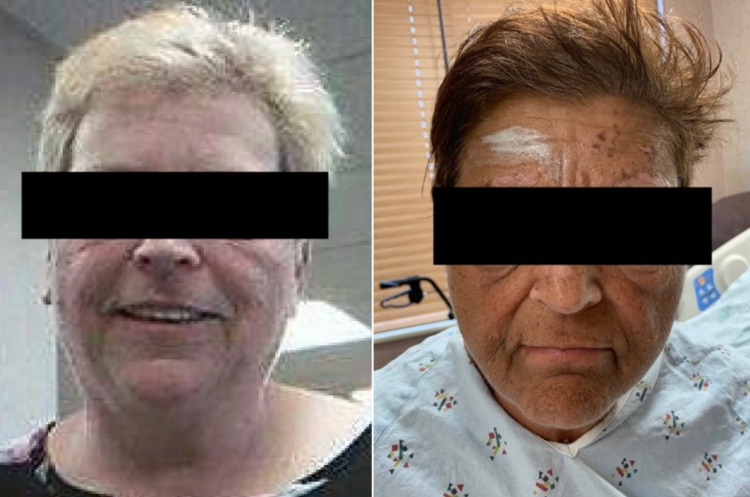
Image from the patient’s chart (left) compared to an image of the patient at presentation (right) demonstrating new darkening of pigmentation.

**Figure 4:**
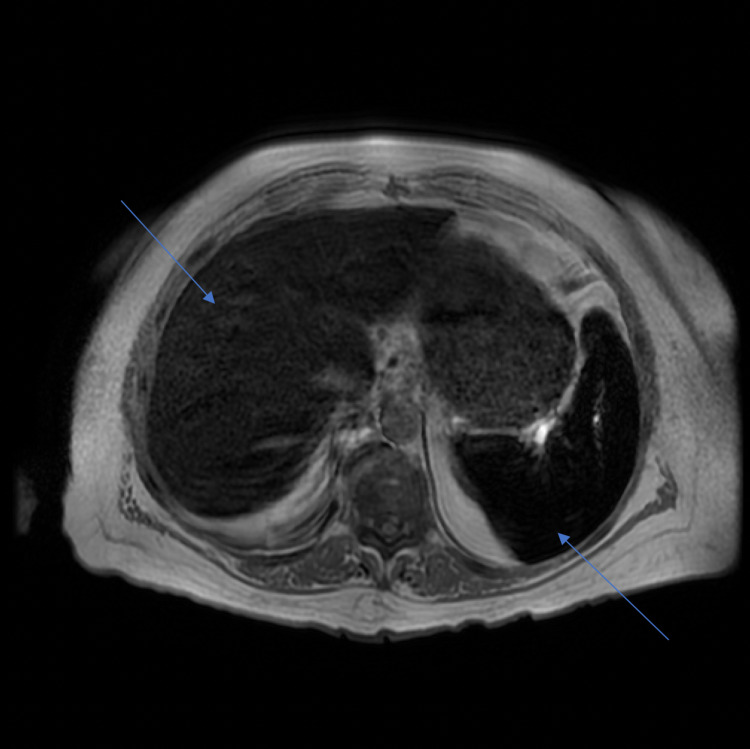
MRI of the abdomen showing iron overload in the liver and spleen. The calculated iron concentration in the liver measured 148 µmol/g (normal iron concentration in the liver is less than 36 µmol/g). MRI: magnetic resonance imaging

A punch biopsy was performed which demonstrated thick homogeneous deposition of immunoglobulin (Ig)G, IgA, and fibrinogen within the walls of superficial dermal vessels (Figure 2). As the biopsy results were concerning for PCT, further workup was ordered which revealed elevated total porphyrins and uroporphyrins (Table 1). On examination, the patient’s pigmentation was noted to be significantly darker compared to a photograph in her chart (Figure 3). Due to the patient’s history of anemia and regular iron infusions every two weeks, previous iron studies were reviewed which showed a high ferritin level and total iron-binding capacity (Table 2). MRI of the liver showed iron overload and steatosis with a calculated iron concentration at 148 µmol/g (Figure 4). Genetic testing failed to demonstrate *C282Y* and *H63D* mutations in the *HFE* gene, supporting a diagnosis of secondary hemochromatosis.

**Table 1:** The patient’s porphyrin studies.

Laboratory study	Value
Serum total porphyrins	253 µg/dL (normal value <0.1 µg/dL)
Uroporphyrin	34.6 µg/dL (normal value <0.1 µg/dL)
Hexacarboxyl porphyrins	2.5 µg/dL (normal value <0.1 µg/dL)
Hepatacarboxyl porphyrins	17 µg/dL (normal value <0.1 µg/dL)
Protoporphyrin	<0.1 µg/dL
Coproporphyrin	<0.1 µg/dL

**Table 2:** The patient’s iron studies.

Laboratory study	Value
Ferritin	5029 ng/mL (H)
Percentage iron saturation	17% (L)
Total iron level	28 µg/dL (L)
Total iron-binding capacity	168 µg/dL (H)

A porphyria genome analysis revealed a rare heterozygous nonsense mutation of the *UROD* gene for a sequence variant designated c.616C>T, which is predicted to result in premature protein termination (p.Gln206*). The mutation itself does not necessarily cause PCT; however, any condition that causes liver damage or iron overload can trigger PCT in the setting of decreased enzyme activity [3]. The patient had received iron infusions with dialysis every two weeks in the 11 months before presentation, with ferritin levels peaking at 5,029 ng/mL two weeks before symptom onset. This provides strong support that iron overload caused by iron infusions for anemia of CKD, with the underlying heterozygous mutation, led to the development of PCT.

The patient was started on hydroxychloroquine 1,000 mg twice weekly. Phlebotomy was not recommended due to the patient’s average hemoglobin level of 8 mg/dL. Her erythropoietin dose was increased to 500 µg twice weekly, and she was started on deferoxamine for iron chelation with the plan to bring her ferritin level down to less than 1,000 ng/mL. Regular iron infusions with hemodialysis per treatment guidelines were discontinued to prevent further iron overload.

## Discussion

PCT is a dermatologic disease marked by skin blistering on sun-exposed areas of the skin (most notably on the dorsal aspects of the hands), skin hyperpigmentation, skin thickening, and facial hypertrichosis. Patients with PCT are also at an increased risk of developing hepatocellular carcinoma [4]. Most cases of PCT are sporadic and caused by viruses and other substances that decrease hepatic function, as described previously; however, 15-50% of PCT cases are caused by familial mutations in the *UROD* gene [5]. Familial PCT is inherited in an autosomal dominant manner, and most patients with familial PCT do not have an affected parent due to reduced penetrance of the gene [6]. The clinical manifestations of PCT in patients with deficient UROD enzyme activity occur when enzyme activity is below 20%. This decrease in enzyme activity occurs when an individual with familial PCT is exposed to other factors that decrease hepatic function such as alcohol, iron overload, and hepatitis C virus. This report highlights a case of familial PCT caused by an iron overload due to regular iron infusions for the treatment of anemia in the setting of CKD. This is the first known case of a patient with a rare heterozygous nonsense mutation of the *UROD* gene at a sequence variant designated c.616C>T.

Patients with CKD are at an increased risk of developing iron-deficiency anemia due to increased levels of hepcidin, as it is a renally cleared peptide [7]. Iron may also be lost during dialysis. Current guidelines recommend regular intravenous iron infusions in ESRD with a maximum allowed ferritin level of 500 ng/mL [8]. Prior to the manifestation of PCT in this patient, she was receiving 4,000 mg of intravenous sodium ferric gluconate complex in sucrose with the dialysis every three months. One month prior to symptom onset, the patient’s ferritin level increased to 5,029 ng/mL. Iron overload alone decreases hepatic UROD enzyme levels, hence, the patient’s iatrogenic iron overload in conjunction with a familial *UROD* mutation likely led to the development of PCT. Interestingly, ESRD has also been shown to be associated with PCT in rare cases likely due to decreased porphyrin excretion [9].

Treatment of PCT in patients with ESRD can be particularly challenging due to chronic anemia that is often associated with the condition. The first-line treatment of PCT traditionally involves phlebotomy with the removal of 450 mL of blood every two weeks and low-dose hydroxychloroquine [10]. However, phlebotomy may be contraindicated in severe cases of anemia, as was the case for this patient. The use of hydroxychloroquine in patients with ESRD may also not be effective due to inefficient porphyrin removal from the blood via dialysis. Hence, iron chelation medications such as deferoxamine have been used in cases of PCT in the setting of ESRD with a successful reduction in ferritin levels [11], as well as erythropoietin to increase hemoglobin level for subsequent phlebotomy. The patient in this case continues to take hydroxychloroquine 100 mg twice weekly and erythropoietin 500 µg every three weeks. Deferoxamine was discontinued due to improvement in ferritin levels and in preparation for therapeutic phlebotomy.

## Conclusions

This case highlights the importance of recognizing the clinical manifestations of PCT, as well as factors, both environmental and iatrogenic, that may lead to its development. Particular care must be taken in patients with a family history of PCT. Treatment and management of PCT may be complicated by concomitant chronic conditions such as ESRD.
